# Topical Application of *Cleome viscosa* Increases the Expression of Basic Fibroblast Growth Factor and Type III Collagen in Rat Cutaneous Wound

**DOI:** 10.1155/2014/680879

**Published:** 2014-04-22

**Authors:** Aadesh Upadhyay, Pronobesh Chattopadhyay, Danswrang Goyary, Papiya M. Mazumder, Vijay Veer

**Affiliations:** ^1^Division of Pharmaceutical Technology, Defence Research Laboratory, Tezpur, Assam 784001, India; ^2^Department of Pharmaceutical Sciences, Birla Institute of Technology, Mesra, Ranchi, Jharkhand 835215, India

## Abstract

*Cleome viscosa* L. (Cleomaceae) is an important traditional medicine of the Indian-Ayurvedic and Chinese-medicine system documented for rheumatic arthritis, hypertension, malaria, neurasthenia, and wound healing. The plant is also known as Asian spider flower and is distributed throughout the greater part of India. The present study explored the wound healing property of *C. viscosa* methanol extract (CvME) and its related mechanism using Wistar rat cutaneous excision wound model. Wound contraction rate, hydroxyproline quantification, and histopathological examination of wound granulation tissue were performed. The healing potential was comparatively assessed with a reference gentamicin sulfate hydrogel (0.01% w/w). Western blot for COL3A1, bFGF, and Smad-2, Smad-3, Smad-4, and Smad-7 was performed with 7-day postoperative granulation tissue. Results revealed that the topical application of CvME (2.5% w/w) significantly accelerated the wound contraction rate (95.14%, 24 postoperative days), increased the hydroxyproline content (3.947 mg/100 mg tissue), and improved histopathology of wound tissue as compared to control groups. Western blot analysis revealed that CvME significantly upregulated the expression of COL3A1 and bFGF and increased the Smad-mediated collagen production in granulation tissue. These findings suggest that *C. viscosa* promoted the wound repair process by attenuating the Smad-mediated collagen production in wound granulation tissue.

## 1. Introduction

Cutaneous wound is a physical injury that results in structural and functional discontinuity of skin tissue, and healing is a tangled tissue restorative process that involves distinct, overlapping phases of homeostasis, inflammation, proliferation, and tissue remodeling [1, 2]. Healing cascade starts from the moment of injury and involves continuous cell-cell and cell-matrix interactions till tissue remodeling of the scar. Blood coagulation at injury site provides temporary fibrin scaffold matrix, which hosts the infiltrating cells and serves as a reservoir of various growth factors and cytokines. Brief initial vasoconstriction at the injury site favors homeostasis and late vasodilation prompts the extravasations of inflammatory cells (neutrophils and macrophages) to remove the nonfunctional cells and destroy the invading infections [2, 3]. Proliferation phase initiated by macrophages involves the formation of granulation tissue and neovascularization. During the final remodeling phase of wound healing, the newly formed collagen fibers cross-link with other structural protein components and contribute to the strength of the scar tissue.

*Cleome viscosa* L. (Cleomaceae) is a sticky herb with yellow flowers, which resembles to mustard, and is distributed throughout the greater part of India. The plant is an important traditional medicine of the Indian-Ayurvedic and Chinese-medicine system for rheumatic arthritis, hypertension, malaria, neurasthenia, and snakebite [4–6]. Various scientific investigations revealed analgesic-antipyretic, anti-inflammatory, hepatoprotective, hypoglycemic, anthelmintic, and antidiarrhoeal activity of* C. viscosa* [4, 7–10]. Panduraju et al. [11] reported the topical application of* C. viscosa* extract accelerated the wound healing in Wistar rat with significant wound contraction rate. Our previous experimental findings showed that methanol extract of* C. viscosa* (CvME) possesses potent antioxidant, antimicrobial, and fibroblast proliferation properties as compared to petroleum ether, chloroform, and water extracts [12]. However, to rationalize the wound healing activity of* C. viscosa,* some major supportive evidences of efficacy such as histopathological examination, estimation of growth factors, and effect on collagen production in healing tissue are yet to be investigated. Therefore, the present study aims to evaluate the* C. viscosa *inducedalteration in fibroblast growth factor and its effect on Smad-mediated collagen (type III) production in Wistar rat excision wound model.

## 2. Materials and Methods

### 2.1. Reagents and Chemicals

Glycine, phenazine methosulfate fluoride (PMSF), Bradford reagent, and Tris-HCl were purchased from Sigma (Sigma Chemical Co., USA). Carbopol 934, propylene glycol, gentamicin sulfate, l-hydroxyproline, and* p*-dimethyl-amino-benzaldehyde were obtained from Himedia (Himedia Pvt. Labs., India). bFGF, COL3A1, Smad-2, Smad-3, Smad-4, and Smad-7, and *β*-actin primary and secondary antibodies and BCIP- (5-bromo-4-chloro-3′-indolyphosphate p-toluidine salt-) NBT reagents were procured from Santa Cruz (Santa Cruz, USA). PVDF membrane was from Millipore (Millipore Corp., USA) and Western Max-HRP-Chromogenic detection kit was purchased from Amresco (Amresco, USA). All other chemicals and reagents not mentioned were of the analytical grade.

### 2.2. Preparation of Extracts

*C. viscosa* leaves were collected during August-September (2011) from the herbal garden of the Defence Research Laboratory, Tezpur, Assam (India), authenticated by the Botanical Survey of India, Shillong (Accession number 085249). About 100 g of shades dried leaf powder was successively extracted using organic solvents (petroleum ether, chloroform, and methanol) by Accelerated Solvent Extractor (ASE 1.5, Dionex, USA) as described earlier [12] and concentrated in a rotary evaporator (Rotavac, Heidolph2, Germany) under reduced pressure. Water extract was freeze dried by lyophilization. Preliminary phytochemical screening was performed as described earlier [13].

### 2.3. Wound Healing Activity

#### 2.3.1. Animals

Healthy adult Swiss albino mice (20–25 g) and Wistar rats (250–300 g) were housed in the Defence Research Laboratory (DRL), Tezpur, Assam, India, and acclimatized for 3 days. Animals were given free access to feed and water* ad libitum*. The experiments were performed according to the Institutional Animal Ethical Committee guidelines (IAEC/DRL/05/July/2011).

#### 2.3.2. Acute Skin Irritation and Toxicity Study

The acute skin irritation and toxicity study was performed as per OECD guidelines-402 (OECD guidelines, 1987) to determine the therapeutic dose of CvME. CvME hydrogel (1 and 2.5% w/w) was applied to the shaved portion at the back of the mice and observed for 14 days for an abnormal skin response, including irritation, redness, itching, and other related symptoms [14].

#### 2.3.3. Animal Grouping and Excision Wound Creation

Rats were inflicted with excision wounds as described by Atiba et al. [15]. Animals were anesthetized and a circular 20 mm diameter wound was created on the shaved dorsal skin up to the depth of loose subcutaneous tissue. Animals were randomly divided into four groups (*n* = 20): group I, nontreated; group II, vehicle control (Carbopol 934 containing 5% propylene glycol); group III, CvME hydrogel treated (2.5% w/w); and group IV, gentamicin sulfate (0.01% w/w). Treatment was given once daily until complete epithelialization. For one-third of animals, wound granulation tissues (excluding any underlying muscle and extraneous tissue) were harvested on the 7th postoperative day. A portion of tissue was processed for Western blotting and another portion was fixed in Histochoice tissue fixative (Amresco, USA) for histopathological evaluations. Half of the remaining animals were euthanized on day 15 after injury; the entire remodeling tissue was used for histopathological assessment and the remaining animals were observed until complete epithelialization.

#### 2.3.4. Wound Contraction Rate and Hydroxyproline Content Estimation

The progressive changes of wounded area were photographed (Nikon Coolpix-S3000) and evaluated by using special size analysis software ImageJ (National Institutes of Health, ImageJ software, downloaded from http://rsb.info.nih.gov/ij/index.html). Wound contraction was expressed as a percentage of the original wound size (day 0).

Hydroxyproline content was analyzed on day 7 in postinjury granulation tissue as described by Neuman and Logan [16]. Acidic tissue hydrolysate was neutralized to pH 7.0 and mixed with 10 mM CuSO_4_ and 2.5 N NaOH followed by 6% H_2_O_2_. The solution was mixed and incubated at 80°C for 5 min with frequent vigorous shaking. Upon cooling, 3 N H_2_SO_4_ was added with agitation. Finally, 5%* p*-dimethyl-amino-benzaldehyde was added and incubated at 70°C for 15 min. Absorbance was measured at 500 nm using a UV-VIS spectrophotometer (CE7200, CECIL, USA). The standard calibration curve was plotted for pure hydroxyproline and used for estimation of the test samples.

#### 2.3.5. Histopathological Evaluation

Tissues were sectioned (6 *μ*m) and stained with hematoxylin-eosin (HE) and Masson's trichrome (MT) stain. Tissue sections were examined for epithelialization, inflammatory cell infiltration, fibroblast proliferation, neovascularization, and collagen deposition.

#### 2.3.6. Western Blot Analysis

The harvested wound tissues were homogenized in lysis buffer (50 mM Tris-HCl, 150 mM NaCl, and 0.05 M PMSF; pH 7.4) and centrifuged at 10,000 ×g for 10 min at 4°C (3-30K, Sigma, Germany). Protein concentration was estimated by Bradford reagent [17]. Equal amount of protein was electrophoresed onto the 12% SDS-PAGE at 80 V for 45 min (Mini Trans-Blot, BioRad Laboratories Inc., USA). Proteins were transblotted on the PVDF membrane (Millipore Corp., USA) and processed with COL3A1, bFGF, Smad-2, Smad-3, Smad-4, and Smad-7, and *β*-actin primary antibodies (1 : 1000) and corresponding secondary antibodies (1 : 2000). The desired proteins were detected by Western Max-HRP-Chromogenic detection kit and BCIP-NBT solution (Amresco, USA).

### 2.4. Statistical Analysis

The results are expressed as means ± standard deviation (S.D.). The treated groups were compared with control groups by analysis of variance following Dunnett's test. A statistical *P* value less than 0.05 was considered significant.

## 3. Results and Discussion

### 3.1. Extraction of Phytochemical from* C. viscosa*

The powdered* C. viscosa* leaf (100 g) yielded 2.82 g inpetroleum ether (CvPE), 1.57 g in chloroform (CvCE), 12.03 g in methanol (CvME), and 9.11 g in water (CvWE). The preliminary phytochemical screening showed the presence of alkaloids, flavonoids, terpenes, and carbohydrates in different extracts. In our earlier study, we have reported about its potent antioxidant, antimicrobial, and human dermal fibroblast proliferation activities of CvME [12] which was similar to the previous reports of Koca et al. [18] and Gouthamchandra et al. [19], where the alcoholic extracts were reported as potent antioxidant and antimicrobial and showed significant wound healing property in comparison to other sequential extracts. Mondal and Suresh [20] also reported the wound healing activity in other species of* Cleome*, where the methanol extract has shown faster wound contraction rate over other extracts. Therefore, CvME was selected for* in vivo* wound healing evaluation using Wistar rat.

### 3.2. Acute Skin Irritation and Toxicity of CvME

Animals treated with CvME hydrogel (1 and 2.5%, w/w) did not show any symptoms of skin irritation and inflammation. Therefore, the extract was considered safe and 2.5% (w/w) of* C. viscosa* methanol extract hydrogel was used for* in vivo* wound healing study.

### 3.3. Effect of CvME on Wound Contraction Rate

**Figure 1:**
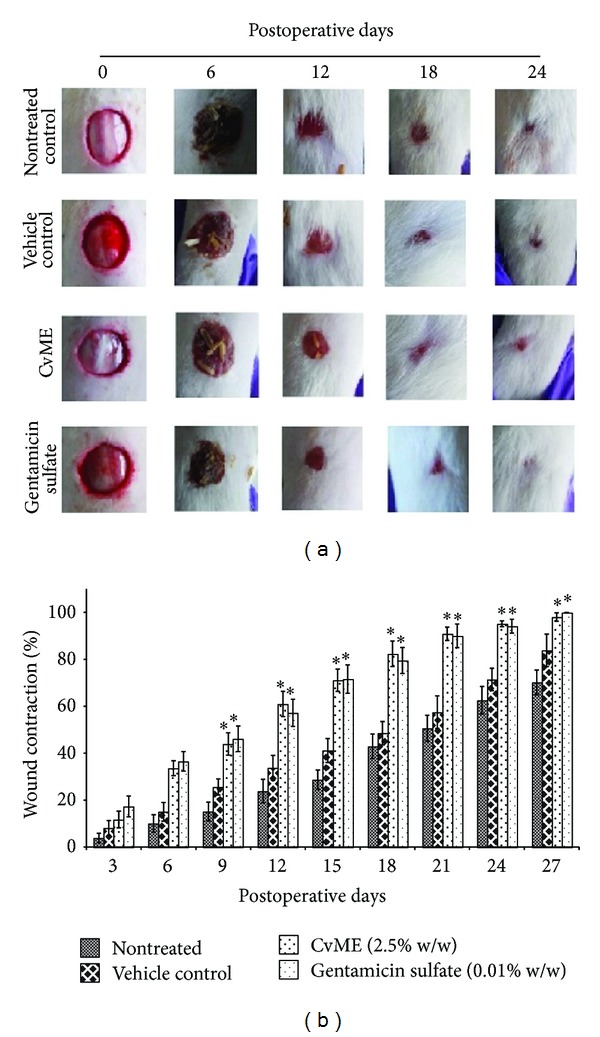
Effect of* C. viscosa* methanol extract (CvME) on wound healing. (a) Pictorial representation of wound closure in Wistar rat; (b) wound contraction rate.* Values are expressed as mean ± SD. Asterisk (∗) indicates significantly different P* < 0.05* as compared to the nontreated and vehicle treated groups*.

Wound contraction is the centripetal movement of surrounding epithelial tissues that depends on the reparative ability and general health of the tissue [21, 22]. The wound appeared clean and free of exudates and distinct granulation tissues appeared at the wound edges from day 3 after injury in CvME and gentamicin sulfate treated groups. Wound areas were reduced parallel to postoperative days and CvME treated group showed 60.99, 71.13, 82.34, and 90.87% wound contraction, whereas gentamicin sulfate treated group showed 57.2, 71.58, 79.51, and 90.02% in 12, 15, 18, and 21 postoperative days, respectively (Figures 1(a) and 1(b)). On the other hand, nontreated control group showed 23.85, 28.74, 42.92, and 50.63% wound contraction and vehicle treated groups showed 33.75, 41.16, 48.54, and 57.49%, in 12, 15, 18, and 21 postoperative days, respectively (Figure 1(b)). Although the wound contraction rate by CvME was comparable to gentamicin sulfate treated groups, it showed significantly higher *P* < 0.05 as compared to nontreated and vehicle treated control groups. These findings coincided with the previous report on the wound healing properties of the methanol extract of* Cleome* species [11, 12, 20]. The difference in wound contraction rate and duration of healing may be due to differences in the experimental and environmental conditions. Many researchers have tried with the methanol extract of herbs/shrubs for wound healing properties to corroborate with specific ethnobotanical knowledge [23]. Similarly, methanol extract was studied and reported to possess better antioxidant, antimicrobial, anti-inflammatory, and wound healing activities than other solvent extracts [12, 18, 19].

### 3.4. Effect of CvME on Hydroxyproline Content

**Figure 2:**
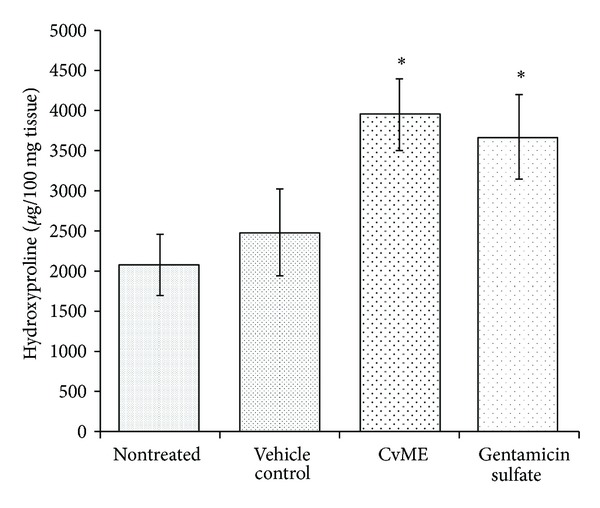
Effect of* C. viscosa* methanol extract on hydroxyproline content of wound granulation tissue.

Hydroxyproline is a basic constituent of collagen structure and contributes to 14% approximately. Measurement of hydroxyproline can be used as an index for collagen turnover [22]. Increase in the hydroxyproline content indicates increased collagen synthesis, which corresponds to the enhanced wound healing. In the present study, the topical application of CvME hydrogel significantly increased (*P* < 0.05) the hydroxyproline content as compared to nontreated and vehicle treated control groups (Figure 2). CvME treatment also showed higher hydroxyproline content as compared to gentamicin sulfate group, although the data were statistically insignificant. These results therefore reflect the potent wound healing activities of CvME in Wistar rats.

### 3.5. Effect of CvME on Histopathology of Wound Granulation Tissue

**Figure 3:**
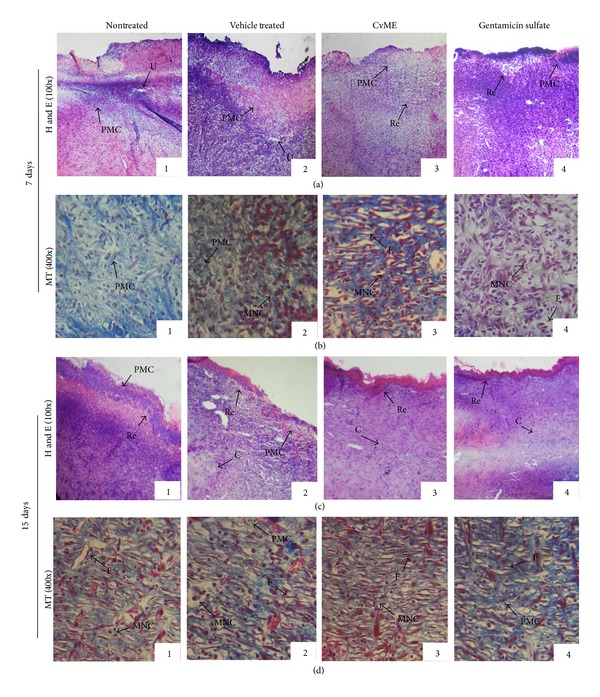
Microscopic view of healing wound tissue and epidermal/dermal remodeling in (1) nontreated, (2) vehicle control, and (3) CvME and (4) gentamicin sulfate treated animal groups. Section shows the hematoxylin and eosin stained epidermis and dermis in (a) and (c) (100x) and Masson's trichrome in (b) and (d) (400x) of 7- and 15-day postoperative treated animal groups, respectively. Arrow points the events of wound healing. S: scab; U: ulcer; Re: reepithelialization; F: fibroblast; PMC: polymorphonuclear cells; MNC: mononuclear cells; C: collagen; and NV: neovascularization.

Table 1 indicated that the histopathology examination of the wound granulation tissue supported the findings of CvME accelerated wound healing as an organized wound repair process (inflammation, proliferation, and remodeling). On the other hand, slow epithelialization rate and less collagen density were observed in vehicle and nontreated groups. Histopathological section of 7-postoperative-day wound tissue showed increased infiltration of macrophages/fibroblasts with less inflammatory cells and ulcer-edematous areas in both CvME and gentamicin sulfate treated groups. On the other hand, mild edema with high polymorphonuclear (inflammatory) cell density and less macrophages/fibroblasts were observed in control groups (Figure 3). Fifteen-day CvME topical application significantly accelerated the cutaneous wound repair processes as evident from the appearance of thick, well organized reepithelialized epidermis and compact dermis with abundant collagen fibers. Masson's trichrome (MT, 400×) staining for 15-day postoperative wound tissue depicted distinct mononuclear cells (macrophage and fibroblast) infiltration and faster keratinization with intraepithelial cornification in CvME (Figure 3), whereas slow reepithelialization and less collagen bundle were noticed in control groups. The results of improved histopathology of wound granulation tissue supported the faster wound contraction rate and elevation of hydroxyproline content in CvME hydrogel treated group, which corroborates the earlier reports on wound healing processes [24–26]. These effects of CvME in wound repair may be due to the presence of phytochemical such as tannins, triterpenoids, flavonoids [4, 27], and their combined effect of antioxidant, antimicrobial, and anti-inflammatory properties [8, 28, 29].

### 3.6. Effect of CvME on Granulation Tissue Protein

**Figure 4:**
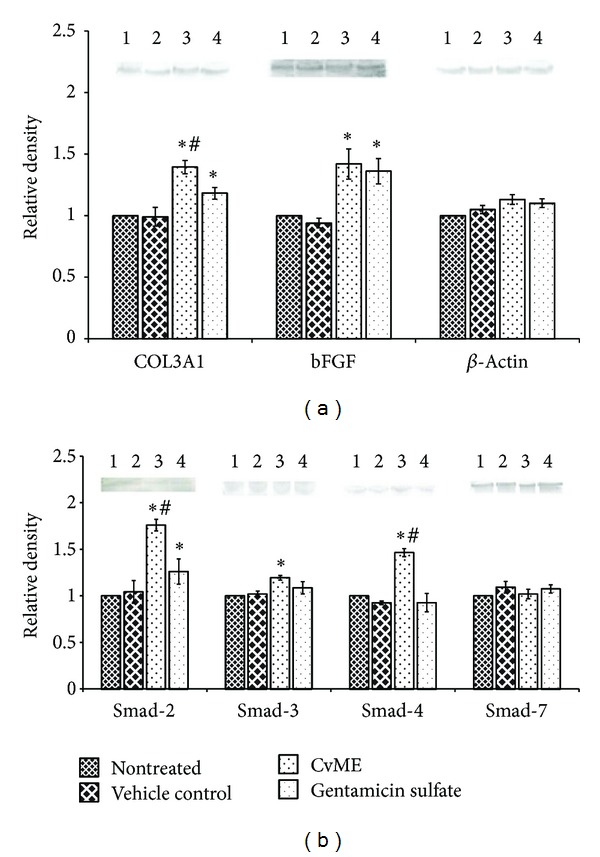
Effect of* C. viscosa* methanol extract (CvME) on COL3A1, bFGF, and Smad-2, Smad-3, Smad-4, and Smad-7 protein expressions on day 7 (seven) in wound tissues, detected by Western blot. Lane (1) represents nontreated, (2) vehicle control, and (3) CvME and (4) gentamicin sulfate treated animal group, respectively.* Values are expressed as mean ± SD. Asterisk (*∗*) indicates significantly different P* < 0.05* as compared to the nontreated and vehicle treated groups*.* Hash (*#*) indicates significantly different P* < 0.05* as compared to gentamicin sulfate treated group.*

Proliferation phase of wound healing involves formation and vascularization of granulation tissue, collagen production, and their subsequent maturation. Collagen type III, present in remodeling wound tissues, is produced by the proliferating young fibroblasts under the influence of various growth factors like transforming growth factor-*β* (TGF-*β*), basic fibroblast growth factor (bFGF), and so forth. The bFGF is a potent angiogenic molecule, which promotes the neovascularization and blood supply to the granulation tissue. Increased oxygen supply facilitates the maturation ofcollagen fibers in granulation tissue [3]. Western blot analysis revealed that application of CvME significantly increased (*P* < 0.05) the expression of COL3A1 and bFGF protein in wound granulation tissue (Figure 4), thus further confirming the above findings of faster wound contraction rate and higher hydroxyproline content.

Various growth factors such as TGF-*β*, bFGF, PDGF (platelet derive growth factor), and VEGF (vascular endothelial growth factor) released by the activated platelets, macrophages, and lymphocytes control the proliferation phase of wound healing [1, 3]. Released TGF-*β* bound to the fibroblast TGF-*β* receptors in granulation tissue and initiates the TGF-*β*-Smad-mediated collagen production cascade [30]. Intracellular Smad family protein (TGF-*β* type I receptor kinases substrate) forms heterocomplex (Smad-2/Smad-3/Smad-4) and transduced the extracellular TGF-*β* signal to fibroblast nucleus for collagen production. Therefore, Smad family proteins were analyzed by Western blot and signal transducer protein (Smad-2, Smad-3, and Smad-4) and showed significantly (*P* < 0.05) higher expression in CvME hydrogel treated group, whereas the inhibitory protein (Smad-7) was observed to be equal in all treatment groups. *β*-Actin was used as an internal control. These findings confirmed that the Smad-mediated mechanism is involved in the increased COL3A1 expression, higher hydroxyproline content, and faster wound contraction rate that contributes to overall improved histopathology during wound repair process.

Therefore, the present study suggested that the topical application of* C. viscose* methanol extractenhances thewound repair process by attenuating TGF*β*-Smad-mediated collagen production in wound granulation tissue.

## Figures and Tables

**Table 1:** Histopathological evaluation of wound healing process in different treatment groups.

Groups	Wound healing process							
	S	U	Ed	PMC	MNC	FP	RE	CD
Day 7								
Nontreated	++	++	++/+	+++	+	+/−	−	−
Vehicle control	+++	++/+	++	+++/++	+	+	−	−/+
CvME (2.5%)	+	−	−	++	++/+++	++	+	++
Gentamicin sulfate (0.01%)	+	−	−	++	++	++	+	++
Day 15								
Nontreated	+	+	−	++	+++	++	+	++
Vehicle control	+	+	−	++	+++	++	++	++
CvME (2.5%)	−	−	−	−/+	+	+++	+++	+++
Gentamicin sulfate (0.01%)	−	−	−	−/+	+	+++	+++	+++

HE and MT staining were scored as mild (+), moderate (++), and severe (+++) for epidermal and/or dermal remodeling. S: scab; U: ulcer; Ed: edema; PMC: polymorphonuclear cells; MNC: mononuclear cells; FP: fibroblast proliferation; CD: collagen deposition; RE: reepithelialization; CvME: *C*. *viscosa* methanol extract.
